# Perceived Stress and Adherence to the Dietary Recommendations and Blood Glucose Levels in Type 1 Diabetes

**DOI:** 10.1155/2020/3548520

**Published:** 2020-07-18

**Authors:** Aila J. Ahola, Carol Forsblom, Valma Harjutsalo, Per-Henrik Groop

**Affiliations:** ^1^Folkhälsan Institute of Genetics, Folkhälsan Research Center, Helsinki, Finland; ^2^Abdominal Center, Nephrology, University of Helsinki and Helsinki University Central Hospital, Helsinki, Finland; ^3^Research Program for Clinical and Molecular Metabolism, Faculty of Medicine, University of Helsinki, Finland; ^4^Chronic Disease Prevention Unit, National Institute for Health and Welfare, Helsinki, Finland; ^5^Department of Diabetes, Central Clinical School, Monash University, Melbourne, Victoria, Australia

## Abstract

Stress may negatively impact self-management of diabetes and thereby deteriorate glycaemic control. Eating is the most frequently reported stress-release method. In this study, we investigated the association between perceived stress (PS), dietary adherence, and glycaemic control. Data from participants in the FinnDiane Study with type 1 diabetes who had completed a diet questionnaire and Cohen's perceived stress scale (PSS) were included. In addition to using a continuous PSS score, participants were divided into three groups based on the PSS scores: the first PSS quartile, low levels of PS; second and third quartiles, moderate levels of PS; and fourth quartile, high levels of PS. A diet score reflecting the level of adherence to dietary recommendations was calculated. Analyses were conducted in the whole sample and in subgroups divided by body mass index (BMI < 25 kg/m^2^ vs. BMI ≥ 25 kg/m^2^). In the whole sample, high PS and continuous stress score were negatively associated with the diet score and with adherence to fish, fresh vegetable, low-fat liquid milk product, and vegetable oil-based cooking fat recommendations. The stress score was negatively associated with the diet score both in lean and in those overweight or obese. However, fish and fresh vegetable recommendations were only affected in those with corpulence. PS was not associated with mean blood glucose concentrations in the whole sample. When divided by BMI status, worse glycaemic control was observed in lean subjects reporting stress. In individuals with overweight or obesity, instead, high glucose concentrations were observed regardless of the level of perceived stress. Interventions to improve stress management could improve dietary adherence and glycaemic control and could thereby have the potential to improve long-term health and well-being of individuals with type 1 diabetes.

## 1. Introduction

Stress is a nonspecific bodily response to any demand made upon it [1]. The detrimental effects of stress are conveyed both directly through neuroendocrine and autonomic responses and indirectly through untoward changes in health behaviours. As adherence to various self-management practices is the cornerstone of daily management of type 1 diabetes, perceived stress could negatively impact the long-term health outcomes of the affected individuals.

Eating is the most frequently reported stress-release method [2]. Importantly, previous research has suggested a change toward unhealthy dietary choices when under stress [3–5], although conflicting results have also been published [6–8]. Studies on the association between stress and eating behaviour in adult individuals with type 1 diabetes are scarce. Over the numerous contacts with healthcare personnel and at diabetes management trainings, the importance of diet on glycaemic control and long-term health is highlighted to individuals with type 1 diabetes. It is therefore likely that these individuals are highly aware of the role that diet plays in their daily management. Stressful life events are, however, prone to happen. It would therefore be important to identify how perceived stress impacts the dietary choices, as well as the blood glucose levels, of individuals with type 1 diabetes.

Our aim was therefore to explore whether there are associations between perceived stress and adherence to dietary recommendations in individuals with type 1 diabetes. We also investigated whether perceived stress is associated with blood glucose concentrations and whether a potential association between perceived stress and diet adherence and glycaemic control differs in lean and overweight or obese participants.

## 2. Materials and Methods

Study subjects were participants in the ongoing nationwide Finnish Diabetic Nephropathy (FinnDiane) Study. The aim of the FinnDiane Study is to identify risk factors for diabetes complications in adult individuals with type 1 diabetes. Type 1 diabetes was defined as diabetes onset before the age of 40 years and permanent insulin treatment initiated within a year from the diagnosis. The Ethics Committee of the Helsinki and Uusimaa Hospital District approved the study protocol. The study was carried out in accordance with the Declaration of Helsinki and its later amendments. Written informed consent was obtained from all individuals prior to study participation.

### 2.1. Clinical Measurements

The procedures conducted at the FinnDiane Study visit have been previously reported [9]. In short, participants' height and weight were measured in light clothing. These measurements were used to calculate body mass index (BMI; kg/m^2^). Glycated haemoglobin (HbA_1c_) was measured locally using a standardized assay. Blood was drawn for subsequent analyses of serum lipid, lipoprotein, and creatinine concentrations at the laboratory of the Helsinki University Hospital. The serum creatinine concentration was used to calculate the estimated glomerular filtration rate (eGFR; ml/min/1.73 m^2^) [10]. The insulin dose was self-reported, and the reported value was used to calculate the dose per body weight (IU/kg).

### 2.2. Perceived Stress

At the study visit, participants were asked to complete a number of questionnaires. Of interest to the current study, a subset of the FinnDiane Study participants filled out Cohen's 14-item perceived stress scale (PSS) [11]. Using a 5-point Likert scale (0 = never, 1 = almost never, 2 = sometimes, 3 = fairly often, and 4 = very often), this questionnaire assesses the extent to which the respondent considers life situations as stressful. After reversing the scores of the positive items, a total score for each individual was obtained by summing up the responses to all the 14 individual items. The total perceived stress score ranges between 0 and 56, with higher scores suggesting higher perceived stress. In the current analyses, the total score was used as a continuous variable. In addition, using a quartile division, we formed three groups. Here, those in the 1^st^ PSS score quartile had low levels of perceived stress; those in the 2^nd^ and 3^rd^ quartiles had moderate levels of perceived stress; and those in the 4^th^ quartile had high levels of perceived stress.

### 2.3. Dietary Intake

Dietary intake was measured with a validated diet questionnaire [12]. In this questionnaire, participants reported their habitual consumption of coffee, tea, liquid milk products, bread, spreads, and cooking fats. Two questions were formulated to assess the habits of salt consumption. Included was also a food frequency questionnaire, where the frequencies of consuming fish dishes, meat dishes, poultry, sausages and cold cuts, legumes, fresh vegetables, cooked vegetables, potatoes, pasta and rice, fruits and berries, high-fat cheese, low-fat cheese, yoghurt and curd, ice cream, soft drinks, sweet pastry, sweets and chocolate, and fried and grilled foods were queried. Here, the frequencies were reported on a 7-level scale (several times a day, once a day, 4-6 times a week, 2-3 times a week, once a week, 1-3 times a month, and less often or never). From the obtained data, we calculated a diet score for each participant. The bases for calculating the diet score have been previously described in detail [13]. In short, each question of the diet questionnaire was compared against the dietary recommendations. For all the food items for which a distinct recommendation was found, participants were scored based on the level of adherence to the recommendation. In all, 11 such food items were identified from the diet questionnaire, and for each of these items, a score ranging from 0 to 2 was given. The total diet score thus ranged between 0 and 22, with the highest scores denoting closer adherence to the recommendations. The total diet score was used as a continuous variable in the analyses. In addition, the individual scores of the 11 food items were also examined. Participants were also asked to complete a three-day food record twice [14]. The recordings were performed with a 10-week interval. In the same record, participants also reported their blood glucose measurements. The mean of these measurements was calculated for each participant and used as a continuous variable in the analyses.

### 2.4. Physical Activity

Participants reported their physical activity in a questionnaire as previously described [15]. In short, for the preceding 12 months, the frequencies, durations, and intensities of the 21 most common types of leisure-time physical activities were reported. For each activity, we calculated an intensity-specific metabolic equivalent of task (MET) as hours per week and summed up all individual MET hours for each participant to obtain the total weekly MET hour. Data on physical activity were used as a covariate in the analyses with the blood glucose concentration as a dependent variable.

### 2.5. Statistical Analyses

All analyses were conducted with IBM SPSS Statistics for Windows, Version 22.0 (IBM Corp, Armonk, NY, USA), and the alpha value was set to 5% (two-tailed test). Descriptive data are presented as frequencies for categorical variables and median (interquartile range) for continuous variables with skewed distribution. Unadjusted analyses to examine between-group differences were conducted with the Chi-squared test and Mann-Whitney *U* test, respectively. Generalized linear regression analysis was used to study the adjusted associations between perceived stress (independent variable) and adherence to the dietary recommendations and blood glucose measurements (dependent variables). The analyses were first conducted in the whole sample comparing individuals with low perceived stress to those with moderate and high perceived stress. We then investigated the associations between perceived stress and the dependent variables separately in individuals with BMI at the range of normal weight (<25 kg/m^2^) and those with overweight or obesity (BMI ≥ 25 kg/m^2^).

## 3. Results

### 3.1. Stress and Dietary Adherence in the Whole Sample

Data were available from 100 individuals (median age 40 years, age range from 25 to 71 years, 51% men). In all, 24 participants reported low levels of perceived stress, while moderate and high levels of perceived stress were reported by 51 and 25 individuals, respectively (Table 1). Apart from individuals with moderate and high levels of perceived stress being younger and having higher eGFR as compared to those with low perceived stress, the groups were comparable with respect to the basic characteristics.

In the unadjusted analyses, compared to those with low perceived stress, individuals with moderate and high perceived stress scored significantly lower in the diet score and its fish and fresh vegetable components (Table 2). In addition, individuals with moderate levels of perceived stress had lower scores for the adherence to vegetable oil-based cooking fats, while those with high perceived stress had lower scores for the adherence to low-fat liquid milk product recommendations.

After adjusting for age, sex, eGFR, and BMI, those with moderate levels of perceived stress had lower scores in adhering to the fresh vegetable recommendations compared to individuals with low perceived stress (Table 3). Instead, those with high perceived stress had a significantly lower diet score and lower scores for its fish, fresh vegetable, low-fat liquid milk product, and vegetable oil-based cooking fat components. After the same adjustments, a higher stress score as a continuous variable was associated with a lower diet score and lower adherence to the recommendations related to the consumption of fish, fresh vegetables, low-fat liquid milk products, and vegetable oil-based cooking fats.

### 3.2. Stress and Dietary Adherence in the BMI Categories

We then investigated whether the association between stress and dietary adherence was modified by being overweight or obese. Compared to the normal-weight participants, individuals with overweight or obesity had comparable perceived stress scores but were older and had lower HDL-cholesterol concentration and higher triglyceride concentration (Table 4).

In the age-, sex-, and eGFR-adjusted models, individuals both with normal weight and overweight or obesity exhibited a negative association between the stress score and the diet score (Table 5). However, the groups divided by BMI were different with respect to the individual components of the diet score that were associated with the stress score. Unlike in those with normal weight, in individuals with overweight or obesity, the perceived stress score was associated with lower adherence to fish and fresh vegetable recommendations. Instead, only those with normal weight exhibited lower adherence to low-fat liquid milk product recommendations with increasing levels of perceived stress. Both BMI groups, however, showed a negative association between adherence to vegetable oil-based cooking fat recommendations and the stress score.

### 3.3. Stress and Blood Glucose Measurements in the Whole Sample

Altogether, 71 participants had reported their blood glucose measurements in a diary. Adjusted for age, sex, eGFR, BMI, physical activity, and insulin dose, individuals with low and moderate levels of perceived stress were comparable with respect to the mean reported blood glucose concentrations (8.0 mmol/l (95% Wald confidence interval 7.3–8.7 mmol/l) vs. 8.7 mmol/l (8.2–9.2 mmol/l), respectively, *p* = 0.131). Comparison between individuals with low and high perceived stress yielded similar results (8.0 mmol/l (7.4–8.7 mmol/l) vs. 8.3 mmol/l (7.6–9.0 mmol/l), respectively, *p* = 0.575).

### 3.4. Stress and Blood Glucose Measurements in the BMI Categories

In all, 31 individuals with normal weight and 40 individuals with overweight or obesity had diary data on the blood glucose concentrations. In normal-weight participants, compared to those with low perceived stress, individuals with moderate perceived stress had significantly higher mean blood glucose concentrations (6.3 mmol/l (4.7–7.9 mmol/l) vs. 9.1 mmol/l (8.4–9.9 mmol/l), respectively, *p* = 0.002), after adjustments for age, sex, eGFR, physical activity, and insulin dose. Similarly, significant differences in blood glucose concentrations were observed in lean individuals between those with low and high perceived stress (6.5 mmol/l (5.6–7.5 mmol/l) vs. 8.4 mmol/l (7.8–9.0 mmol/l), respectively, *p* = 0.004). In those with overweight or obesity, instead, the level of glycaemia was no different between those with low and moderate perceived stress (8.4 mmol/l (7.7–9.0 mmol/l) vs. 8.3 mmol/l (7.7–8.8 mmol/l), respectively, *p* = 0.780) and between those with low and high perceived stress (8.6 mmol/l (8.0–9.3 mmol/l) vs. 7.9 mmol/l (7.0–8.8 mmol/l), respectively, *p* = 0.262).

## 4. Discussion

In the current study, high perceived stress was significantly associated with less healthy diet judged by lower adherence to the dietary recommendations. In particular, the scores reflecting the level of adherence to fish, fresh vegetable, low-fat liquid milk products, and vegetable oil-based cooking fat recommendations were lower in individuals reporting high levels of stress. We also investigated whether the association between stress and dietary adherence would be modified by overweight or obesity. While stress was observed to reduce the adherence to the overall dietary recommendations in both lean and those with corpulence, there seemed to be some differences in which individual recommendations were affected in these two groups. Notably, unlike in lean individuals, stress was associated with reduced adherence to fish and fresh vegetable recommendations in those with overweight or obesity. Finally, we investigated the association between perceived stress and glycaemia. Here, stress was associated with higher levels of mean blood glucose levels only in lean participants. In individuals with overweight or obesity, instead, high glucose concentrations were observed regardless of the level of perceived stress.

While we are not aware of other studies on perceived stress and adherence to the dietary recommendations in adults with type 1 diabetes, the association has been investigated in a number of other populations. Among these is the study in 5077 adults by Isasi et al., who observed an inverse association between perceived stress and a healthy eating index suggesting a less healthy eating pattern among those reporting higher perceived stress during the past month [3]. Similarly, among 1189 university students in Finland, perceived stress was negatively associated with a dietary guideline adherence index [4]. Moreover, compared to the normal-weight students, this association was more pronounced in those with overweight. An inverse relationship between the level of perceived stress and the overall diet quality was also observed in a sample of European adolescents [5]. In addition to the above-mentioned observations showing an adverse association between stress and diet quality, a number of studies with no association have also been published. Among these is the study by Carson et al., who investigated the relationship between perceived stress and meeting of food group recommendations in 355 African American women [6]. In their study, neither stress score nor grouping of participants based on the median stress score was associated with meeting the recommendations. Similarly, in a large sample of university students in Egypt, no association was observed between stress and an index of dietary guideline adherence [7]. In a sample of overweight and obese working-age Finns, again, the adherence to a healthy diet was no different among the tertiles of perceived stress [8]. However, a higher level of perceived stress was associated with lower intuitive eating, lower eating competence, and lower cognitive restraints. Moreover, perceived stress was positively associated with uncontrolled and emotional eating and a higher tendency to use food as a reward.

In addition to the studies investigating the association between perceived stress and adherence to the overall dietary recommendations, a number of studies have also reported the association between stress and individual food items. Here, similar to the observations made in the current study, perceived stress was associated with reduced consumption of fruits and vegetables in some studies [7, 16, 17] but not all [18]. A number of studies have also highlighted the positive association between perceived stress and consumption of sweets [17–19]. However, in line with the observations by El Ansari et al. [4, 7], no such association was evident in the current study. This lack of association between stress and the consumption of sweet food items, in the current study, may partly be explained by the population in question. After all, the importance of avoiding sweet foods in controlling blood glucose fluctuations in type 1 diabetes is likely acknowledged regardless of the participants' stress level. Yet, a number of studies have linked perceived stress with higher intake of fast foods or snacks [16–18, 20]. Preference for quickly available meals when stressed is in concordance with the reports that perceived stress is also associated with haphazard meal planning [21]. During stressful periods, individual may, therefore, be less likely to plan one's meals carefully. While “fast foods,” as an individual food item, were not included in our questionnaire, we did observe that perceived stress was associated with lower adherence to the recommendations related to fish, fresh vegetables, low-fat liquid milk products, and vegetable oil-based cooking fats, that is, food items that may be related to properly planned meals. Of interest, the association between perceived stress and haphazard meal planning has shown to be particularly pronounced in individuals with overweight or obesity [21]. In line with these observations, when we divided the study population by the BMI status, perceived stress was associated with reduced adherence with fish and fresh vegetable recommendations only in subjects with overweight or obesity.

Using functional magnetic resonance, Tyron et al. observed that, in participants reporting higher chronic stress, pictures of foods high in energy content elicited exaggerated activity in regions of the brain involving reward, motivation, and habitual decision-making [22]. Moreover, viewing the same pictures of high-energy foods was associated with significant deactivation of frontal brain regions that are linked to strategic planning and emotional control. These results suggest that stress may alter the brain's response to foods in ways that predispose individuals to unhealthy dietary choices. While poor stress management has been associated with worse health risk behaviour profile, it is noteworthy that health behaviour change intervention targeted to improve stress management skills has also been associated with improvements in health behaviours [23].

Stress may impair glucose metabolism through dysregulation of the hypothalamic-pituitary-adrenal and sympathetic-adrenal-medullary axes [24]. Indeed, acute stress activates central, autonomic, neuroendocrine, and immune systems in order to harness the individual with sufficient fuel to either fight or flight in the face of the stressor. In such a state, insulin resistance also occurs. Subsequently, it would be reasonable to expect increased blood glucose levels upon experiencing stress. However, in the current study, perceived stress was not in the whole population associated with the daily mean of the blood glucose measurements. In line with our observations, Halford et al. reported that only 7 out of 15 patients exhibited increased blood glucose levels when stressed and concluded that stress influenced blood glucose levels at least in some individuals with diabetes [25]. Similarly, Kramer et al. encountered major heterogeneity in the association between stress and blood glucose concentrations in individuals with diabetes, as less than half of the participants showed any such association [26]. Of interest, when investigating the association between stress and glycaemia, Ozaslan et al. noted that the blood glucose-rising effect of stress was diminished by increasing BMI [27]. Our observations with higher blood glucose concentrations in lean subjects reporting stress and no association between perceived stress and glycaemia in overweight or obese subjects are in line with the reports by Ozaslan et al., suggesting that additional factors impact glucose dynamics under stress. Indeed, prevailing insulin resistance, related to obesity, may mask the association between perceived stress and glycaemia [28].

Well-defined study participants with objectively measured height and weight are important strengths of this study. The study population was, however, small. Due to the small sample size, we could not, for example, investigate the sex differences in the perceived stress and dietary adherence. This may be important, as 68% of those reporting high levels of stress were women, compared to 50% of those with low perceived stress. Larger studies are needed to further elaborate such potential differences. Another limitation is the use of self-reported methods to study dietary intake, blood glucose values, and stress. Importantly, incorporating a physiologic measurement of stress, such as measuring salivary cortisol, would have reduced the measurement error related to subjective assessment. Finally, due to the observational nature of the study, no causal relationships can be determined.

## 5. Conclusions

Perceived stress was associated with reduced adherence to dietary recommendations in participants with type 1 diabetes: fish, fresh vegetable, low-fat liquid milk product, and vegetable oil-based cooking fat recommendations, in particular. Moreover, while in overweight or obese individuals, high blood glucose concentrations were observed regardless of the stress level; stress perception in lean participants was observed to impair the glycaemic control. Interventions to improve stress management could improve dietary adherence and glycaemic control and could thereby have the potential to improve long-term health and well-being of individuals with type 1 diabetes.

## Figures and Tables

**Table 1:** Basic characteristics divided by perceived stress.

	Low stress	Moderate stress	High stress
	*n* = 24	*n* = 51	*n* = 25
Stress score	11 (7–12)	19 (17–22)^∗∗∗^	37 (30–41)^∗∗∗^
Men (%)	50.0	60.8	32.0
Age (years)	41 (38–45)	40 (33–46)^∗^	35 (31–43)^∗^
BMI (kg/m^2^)	26.6 (25.1–29.0)	25.1 (23.3–29.9)	26.3 (23.3–30.4)
eGFR (ml/min/1.73 m^2^)	107 (91–113)	109 (97–119)^∗^	115 (107–119)^∗^
HbA_1c_ (mmol/mol)	54 (51–67)	64 (55–69)	59 (53–65)
HbA_1c_ (%)	7.1 (6.8–8.3)	8.0 (7.2–8.5)	7.5 (7.0–8.1)
Cholesterol (mmol/l)	4.49 (3.94–5.03)	4.35 (3.95–4.92)	4.51 (4.06–5.24)
HDL-cholesterol (mmol/l)	1.23 (1.00–1.52)	1.42 (1.15–1.76)	1.40 (1.16–1.76)
Triglycerides (mmol/l)	1.0 (0.8–1.4)	1.0 (0.7–1.6)	0.8 (0.7–1.6)

Data are presented as the median (interquartile range) for continuous variables as they had skewed distribution and frequency for categorical variables. Between-group comparisons were conducted with the Mann-Whitney *U* test and Chi-squared test, respectively. In the comparisons, the moderate and high perceived stress groups were compared to the group with low perceived stress. Low stress: 1^st^ quartile of the perceived stress scale; moderate stress: 2^nd^ and 3^rd^ quartiles of the perceived stress scale; high stress: 4^th^ quartile of the perceived stress scale; BMI: body mass index. ^∗^*p* < 0.05; ^∗∗∗^*p* < 0.001.

**Table 2:** Diet score divided by perceived stress.

	Low stress	Moderate stress	High stress
	*n* = 24	*n* = 51	*n* = 25
Diet score	12 (9–14)	10 (9–12)^∗^	9 (6.5–10)^∗∗^
Components of the diet score			
Fish	1 (1–2)	1 (0–1)^∗^	0.5 (0–1)^∗^
Fresh vegetables	2 (1–2)	1 (0–1)^∗∗^	1 (0–1)^∗^
Cooked vegetables	1.5 (0–2)	1 (0–2)	1 (0–2)
Fruits and berries	1 (0–2)	1 (0–1)	0 (0–1.5)
Sweet pastry	1.5 (0–2)	2 (1–2)	1 (0.5–2)
Sweets and chocolate	0.5 (0–2)	1 (0–2)	0 (0–1)
Soft drinks	1 (0–2)	1 (0–2)	1 (0–1)
Low-fat liquid milk products	0.5 (0–2)	0 (0–1)	0 (0–1)^∗^
Vegetable oil spreads	0 (0–2)	0 (0–2)	0 (0–2)
Vegetable oil cooking fats	2 (2–2)	2 (0–2)^∗∗^	2 (0–2)
Salt	0 (0–1)	0 (0–1)	0 (0–1)

Data are presented as the median (interquartile range). Between-group comparisons were conducted with the Mann-Whitney *U* test. In the comparisons, the moderate and high perceived stress groups were compared to the group with low perceived stress. Low stress: 1^st^ quartile of the perceived stress scale; moderate: 2^nd^ and 3^rd^ quartiles of the perceived stress scale; high stress: 4^th^ quartile of the perceived stress scale. ^∗^*p* < 0.05; ^∗∗^*p* < 0.01.

**Table 3:** The multivariable association between perceived stress and diet score.

	Low stress	Moderate stress		Low stress	High stress		Stress score	
	Mean (95 Wald CI)	Mean (95 Wald CI)	*p*	Mean (95 Wald CI)	Mean (95 Wald CI)	*p*	*B* (95% Wald CI)	*p*
Diet score	11.5 (10.3–12.7)	10.0 (9.2–10.9)	0.056	11.3 (10.0–12.5)	9.1 (7.8–10.3)	0.016	-0.10 (-0.16–-0.05)	0.001
Components of the diet score								
Fish	1.2 (0.9–1.5)	0.8 (0.6–1.0)	0.068	1.2 (0.9–1.5)	0.7 (0.4–0.9)	0.018	-0.02 (-0.03–-0.01)	0.029
Fresh vegetables	1.3 (1.0–1.6)	0.8 (0.5–1.0)	0.003	1.3 (1.0–1.6)	0.8 (0.5–1.1)	0.010	-0.02 (-0.03–-0.01)	0.021
Cooked vegetables	1.1 (0.8–1.5)	1.0 (0.8–1.2)	0.561	1.2 (0.8–1.6)	1.1 (0.7–1.5)	0.761	0.00 (-0.02–0.02)	0.844
Fruits and berries	0.9 (0.6–1.2)	0.7 (0.5–0.9)	0.461	0.9 (0.6–1.3)	0.7 (0.4–1.1)	0.467	-0.01 (-0.02–0.01)	0.488
Sweet pastry	1.2 (0.9–1.5)	1.5 (1.3–1.7)	0.083	1.1 (0.8–1.4)	1.2 (0.9–1.6)	0.590	-0.01 (-0.02–0.01)	0.622
Sweets and chocolate	0.9 (0.5–1.2)	1.0 (0.7–1.2)	0.655	0.8 (0.4–1.1)	0.8 (0.5–1.1)	0.894	-0.01 (-0.02–0.01)	0.403
Soft drinks	1.0 (0.7–1.3)	1.0 (0.8–1.2)	0.946	1.0 (0.7–1.3)	0.9 (0.6–1.2)	0.692	0.00 (-0.01–0.02)	0.922
Low-fat liquid milk products	0.8 (0.5–1.1)	0.5 (0.3–0.7)	0.143	0.8 (0.5–1.1)	0.3 (0.0–0.6)	0.014	-0.02 (-0.03–-0.01)	0.012
Vegetable oil spreads	0.8 (0.4–1.2)	0.9 (0.6–1.2)	0.683	0.7 (0.3–1.1)	0.8 (0.4–1.2)	0.866	-0.01 (-0.02–0.01)	0.593
Vegetable oil cooking fats	1.8 (1.4–2.1)	1.5 (1.3–1.7)	0.220	1.8 (1.5–2.1)	1.3 (1.0–1.6)	0.033	-0.03 (-0.04–-0.01)	0.001
Salt	0.6 (0.3–0.9)	0.4 (0.2–0.6)	0.366	0.5 (0.3–0.8)	0.4 (0.2–0.7)	0.674	-0.01 (-0.02–0.01)	0.476

Generalized linear regression analysis. CI: confidence interval. All models are adjusted for age, sex, estimated glomerular filtration rate, and BMI. Low stress: 1^st^ quartile of the perceived stress scale; moderate stress: 2^nd^ and 3^rd^ quartiles of the perceived stress scale; high stress: 4^th^ quartile of the perceived stress scale.

**Table 4:** Basic characteristics divided by BMI classification.

	BMI < 25 kg/m^2^	BMI ≥ 25 kg/m^2^	
	*n* = 42	*n* = 58	*p*
Stress score	21 (17–27)	17 (13–25)	0.212
Men (%)	47.6	53.4	0.686
Age (years)	37 (31–43)	41 (37–45)	0.047
BMI (kg/m^2^)	23.3 (21.8–24.5)	28.4 (26.7–31.7)	<0.001
eGFR (ml/min/1.73 m^2^)	111 (102–118)	109 (93–117)	0.194
HbA_1c_ (mmol/mol)	57 (52–67)	63 (54–67)	0.170
HbA_1c_ (%)	7.4 (6.9–8.3)	7.9 (7.1–8.3)	0.170
Cholesterol (mmol/l)	4.36 (4.02–4.60)	4.54 (3.99–5.12)	0.271
HDL-cholesterol (mmol/l)	1.49 (1.27–1.87)	1.27 (1.03–1.58)	0.018
Triglycerides (mmol/l)	0.8 (0.7–1.1)	1.1 (0.8–2.0)	0.002

Data are presented as the median (interquartile range) for continuous variables as they had skewed distribution and frequency for categorical variables. Between-group comparisons were conducted with the Mann-Whitney *U* test and Chi-squared test, respectively. BMI: body mass index.

**Table 5:** The multivariable association between perceived stress score and diet score divided by BMI classification.

	BMI < 25 kg/m^2^		BMI ≥ 25 kg/m^2^	
	*B* (95% Wald CI)	*p*	*B* (95% Wald CI)	*p*
Diet score	-0.17 (-0.27–-0.06)	0.002	-0.07 (-0.14–-0.01)	0.022
Components of the diet score				
Fish	0.01 (-0.03–0.03)	0.760	-0.02 (-0.04–-0.01)	0.002
Fresh vegetables	-0.01 (-0.04–0.02)	0.373	-0.02 (-0.04–-0.01)	0.023
Cooked vegetables	0.00 (-0.03–0.03)	0.985	0.00 (-0.02–0.02)	0.880
Fruits and berries	0.00 (-0.02–0.03)	0.949	-0.01 (-0.03–0.01)	0.341
Sweet pastry	-0.01 (-0.04–0.01)	0.303	0.00 (-0.02–0.02)	0.964
Sweets and chocolate	-0.02 (-0.05–0.01)	0.115	0.00 (-0.02–0.02)	0.938
Soft drinks	-0.02 (-0.04–0.00)	0.089	0.01 (-0.01–0.03)	0.326
Low-fat liquid milk products	-0.02 (-0.05–-0.01)	0.043	-0.02 (-0.03–0.00)	0.088
Vegetable oil spreads	-0.03 (-0.06–0.00)	0.071	0.01 (-0.02–0.03)	0.657
Vegetable oil cooking fats	-0.03 (-0.06–-0.01)	0.039	-0.02 (-0.04–-0.01)	0.010
Salt	-0.02 (-0.04–0.00)	0.053	0.00 (-0.01–0.02)	0.663

Generalized linear regression analysis. BMI: body mass index; CI: confidence interval. All models are adjusted for age, sex, and estimated glomerular filtration rate.

## Data Availability

The data used to support the findings of this study are available from the corresponding authors upon request.

## Supplementary Materials

Supplementary materials List of physicians and nurses participating in the enrollment of patients.
