# Supplementary material for: Perceived Stress and Adherence to the Dietary Recommendations and Blood Glucose Levels in Type 1 Diabetes
Source: J Diabetes Res. 2020 Jul 18;2020:3548520. doi: 10.1155/2020/3548520 (PMC7383306; doi:10.1155/2020/3548520)
Supplement: Supplementary materials — List of physicians and nurses participating in the enrollment of patients. [file 3548520.f1.pdf]

Perceived stress and adherence to the dietary recommendations and blood glucose levels in type 1 diabetes

Aila J. Ahola,[1,2,3] Carol Forsblom,[1,2,3] Valma Harjutsalo,[1,2,3,4] Per-Henrik Groop,[1,2,3,5]*, on behalf of the FinnDiane Study Group

[1]Folkhälsan Institute of Genetics, Folkhälsan Research Center, Helsinki, Finland

[2]Abdominal Center, Nephrology, University of Helsinki and Helsinki University Central Hospital, Helsinki, Finland

[3]Research Program for Clinical and Molecular Metabolism, Faculty of Medicine, University of Helsinki, Finland

[4]Chronic Disease Prevention Unit, National Institute for Health and Welfare, Helsinki, Finland

[5]Department of Diabetes, Central Clinical School, Monash University, Melbourne, Victoria, Australia.

*Corresponding author: Per-Henrik Groop, Folkhälsan Research Center, Biomedicum Helsinki C318b, PO Box 63, FI-00014 University of Helsinki, Finland. Tel +358 500 430 436. Email: per-henrik.groop@helsinki.fi

The Finnish Diabetic Nephropathy Study Centers

Anjalankoski Health Center	S.Koivula, T.Uggeldahl
Central Finland Central Hospital, Jyväskylä	T.Forslund, A.Halonen, A.Koistinen, P.Koskiaho, M.Laukkanen, J.Saltevo, M.Tiihonen
Central Hospital of Åland Islands, Mariehamn	M.Forsen, H.Granlund, A.-C.Jonsson, B.Nyroos
Central Hospital of Kanta-Häme, Hämeenlinna	P.Kinnunen, A.Orvola, T.Salonen, A.Vähänen
Central Hospital of Kymenlaakso, Kotka	R.Paldanius, M.Riihelä, L.Ryysy
Central Hospital of Länsi-Pohja, Kemi	H.Laukkanen, P.Nyländen, A.Sademies
Central Ostrobothnian Hospital District, Kokkola	S.Anderson, B.Asplund, U.Byskata, P.Liedes, M.Kuusela, T.Virkkala
City of Espoo Health Center:	
Espoonlahti	A.Nikkola, E.Ritola
Tapiola	M.Niska, H.Saarinen
Samaria	E.Oukko-Ruponen, T.Virtanen
Viherlaakso	A.Lyytinen
City of Helsinki Health Center:	
Puistola	H.Kari, T.Simonen
Suutarila	A.Kaprio, J.Kärkkäinen, B.Rantaeskola
Töölö	P.Kääriäinen, J.Haaga, A-L.Pietiläinen
City of Hyvinkää Health Center	S.Klemetti, T.Nyandoto, E.Rontu, S.Satuli-Autere
City of Vantaa Health Center:	
Korso	R.Toivonen, H.Virtanen
Länsimäki	R.Ahonen, M.Ivaska-Suomela, A.Jauhiainen
Martinlaakso	M.Laine, T.Pellonpää, R.Puranen
Myyrmäki	A.Airas, J.Laakso, K.Rautavaara
Rekola	M.Erola, E.Jatkola
Tikkurila	R.Lönnblad, A.Malm, J.Mäkelä, E.Rautamo
Heinola Health Center	P.Hentunen, J.Lagerstam
Helsinki University Central Hospital, Department of Medicine, Division of Nephrology	M.Feodoroff, D.Gordin, O.Heikkilä, K.Hietala, J.Fagerudd, M.Korolainen, L.Kyllönen, J.Kytö, S.Lindh, K.Pettersson-Fernholm, M.Rosengård-Bärlund, A.Sandelin, L.Thorn, J.Tuomikangas, T.Vesisenaho, J.Wadén
Herttoniemi Hospital, Helsinki	V.Sipilä
Hospital of Lounais-Häme, Forssa	T.Kalliomäki, J.Koskelainen, R.Nikkanen, N.Savolainen, H.Sulonen, E.Valtonen
Hyvinkää Hospital	L. Norvio, A.Hämäläinen
Iisalmi Hospital	E.Toivanen
Jokilaakso Hospital, Jämsä	A.Parta, I.Pirttiniemi
Jorvi Hospital, Helsinki University Central Hospital	S.Aranko, S.Ervasti, R.Kauppinen-Mäkelin, A.Kuusisto, T.Leppälä, K.Nikkilä, L.Pekkonen
Jyväskylä Health Center, Kyllö	K.Nuorva, M.Tiihonen
Kainuu Central Hospital, Kajaani	S.Jokelainen, K.Kananen, M.Karjalainen, P.Kemppainen, A-M.Mankinen,
A.Reponen	M.Sankari
Kerava Health Center	H.Stuckey, P.Suominen
Kirkkonummi Health Center	A.Lappalainen, M.Liimatainen, J.Santaholma
Kivelä Hospital, Helsinki	A.Aimolahti, E.Huovinen
Koskela Hospital, Helsinki	V.Ilkka, M.Lehtimäki
Kotka Health Center	E.Pälikkö-Kontinen, A.Vanhanen
Kouvola Health Center	E.Koskinen, T.Siitonen
Kuopio University Hospital	E.Huttunen, R.Ikäheimo, P.Karhapää, P.Kekäläinen, M.Laakso, T.Lakka, E.Lampainen, L.Moilanen, S. Tanskanen L.Niskanen, U.Tuovinen, I.Vauhkonen, E.Voutilainen
Kuusamo Health Center	T.Kääriäinen, E.Isopoussu
Kuusankoski Hospital	E.Kilkki, I.Koskinen, L.Riihelä
Laakso Hospital, Helsinki	T.Meriläinen, P.Poukka, R.Savolainen, N.Uhlenius
Lahti City Hospital	A.Mäkelä, M.Tanner
Lapland Central Hospital, Rovaniemi	L.Hyvärinen, K.Lampela, S.Pöykkö, T.Rompasaari, S.Severinkangas, T.Tulokas
Lappeenranta Health Center	P. Erola, L.Härkönen, P.Linkola, T.Pekkanen, I.Pulli, E.Repo
Lohja Hospital	T.Granlund, K.Hietanen, M.Porrassalmi, M.Saari, T.Salonen, M.Tiikkainen,
Länsi-Uusimaa Hospital, Tammisaari	I.-M.Jousmaa, J.Rinne
Loimaa Health Center	A.Mäkelä, P.Eloranta
Malmi Hospital, Helsinki	H.Lanki, S.Moilanen, M.Tilly-Kiesi
Mikkeli Central Hospital	A.Gynther, R.Manninen, P.Nironen, M.Salminen, T.Vänttinen
Mänttä Regional Hospital	I.Pirttiniemi, A-M.Hänninen
North Karelian Hospital, Joensuu	U-M.Henttula, P.Kekäläinen, M.Pietarinen, A.Rissanen, M.Voutilainen
Nurmijärvi Health Center	A.Burgos, K.Urtamo
Oulaskangas Hospital, Oulainen	E.Jokelainen, P-L.Jylkkä, E.Kaarlela, J.Vuolaspuro
Oulu Health Center	L.Hiltunen, R.Häkkinen, S.Keinänen-Kiukaanniemi
Oulu University Hospital	R.Ikäheimo

Päijät-Häme Central Hospital	H.Haapamäki, A.Helanterä, S.Hämäläinen, V.Ilvesmäki, H.Miettinen
Palokka Health Center	P.Sopanen, L.Welling
Pieksämäki Hospital	V.Sevtsenko, M.Tamminen
Pietarsaari Hospital	M-L.Holmbäck, B.Isomaa, L.Sarelin
Pori City Hospital	P.Ahonen, P.Merisalo, E.Muurinen, K.Sävelä
Porvoo Hospital	M.Kallio, B.Rask, S.Rämö
Raahe Hospital	A.Holma, M.Honkala, A.Tuomivaara, R.Vainionpää
Rauma Hospital	K.Laine, K.Saarinen, T.Salminen
Riihimäki Hospital	P.Aalto, E.Immonen, L.Juurinen
Salo Hospital	A.Alanko, J.Lapinleimu, P.Rautio, M.Virtanen
Satakunta Central Hospital, Pori	M.Asola, M.Juhola, P.Kunelius, M.-L.Lahdenmäki, P.Pääkkönen, M.Rautavirta
Savonlinna Central Hospital	T.Pulli, P.Sallinen, M.Taskinen, E.Tolvanen, T.Tuominen H.Valtonen, A.Vartia, S-L.Viitanen
Seinäjoki Central Hospital	O.Antila, E.Korpi-Hyövälti, T.Latvala, E.Leijala, T.Leikkari, M.Punkari N.Rantamäki, H.Vähävuori
South Karelia Central Hospital, Lappeenranta	T.Ensala, E.Hussi, R.Härkönen, U.Nyholm, J.Toivanen
Tampere Health Center	A.Vaden, P.Alarotu, E.Kujansuu, H.Kirkkopelto-Jokinen, M.Helin, S.Gummerus, L.Calonius, T.Niskanen, T.Kaitala, T.Vatanen
Tampere University Hospital	P. Hannula, I.Ala-Houhala, R.Kannisto, T.Kuningas, P.Lampinen, M.Määttä, H.Oksala, T.Oksanen, A.Putila, H.Saha, K.Salonen, H.Tauriainen, S.Tulokas
Tiirismaa Health Center, Hollola	T.Kivelä, L.Petlin, L.Savolainen
Turku Health Center	A.Artukka, I.Hämäläinen, L.Lehtinen, E.Pyysalo, H.Virtamo, M.Viinikkala, M.Vähätalo
Turku University Central Hospital	K.Breitholz, R.Eskola, K.Metsärinne, U.Pietilä, P.Saarinen, R.Tuominen, S.Äyräpää
Vaajakoski Health Center	K.Mäkinen, P.Sopanen
Valkeakoski Regional Hospital	S.Ojanen, E.Valtonen, H.Ylönen, M.Rautiainen, T.Immonen
Vammala Regional Hospital	I.Isomäki, R.Kroneld, L.Mustaniemi, M.Tapiolinna-Mäkelä
Vasa Central Hospital	S.Bergkulla, U.Hautamäki, V-A.Myllyniemi, I.Rusk
